# Supplementary material for: Modulating cortical excitability and cortical arousal by pupil self-regulation
Source: Nat Commun. 2025 May 16;16:4552. doi: 10.1038/s41467-025-59837-5 (PMC12084627; doi:10.1038/s41467-025-59837-5)
Supplement: Supplementary file 2 — Reporting Summary [file 41467_2025_59837_MOESM2_ESM.pdf]

nature portfolio

Corresponding author(s): Marieke Lieve Weijs, Nicole Wenderoth

Last updated by author(s): Apr 24, 2025

Reporting Summary

Nature Portfolio wishes to improve the reproducibility of the work that we publish. This form provides structure for consistency and transparency in reporting. For further information on Nature Portfolio policies, see our Editorial Policies and the Editorial Policy Checklist.

Statistics

For all statistical analyses, confirm that the following items are present in the figure legend, table legend, main text, or Methods section.

n/a	Confirmed	
☐	☒	The exact sample size (*n*) for each experimental group/condition, given as a discrete number and unit of measurement
☐	☒	A statement on whether measurements were taken from distinct samples or whether the same sample was measured repeatedly
☐	☒	The statistical test(s) used AND whether they are one- or two-sided<br>*Only common tests should be described solely by name; describe more complex techniques in the Methods section.*
☐	☒	A description of all covariates tested
☐	☒	A description of any assumptions or corrections, such as tests of normality and adjustment for multiple comparisons
☐	☒	A full description of the statistical parameters including central tendency (e.g. means) or other basic estimates (e.g. regression coefficient) AND variation (e.g. standard deviation) or associated estimates of uncertainty (e.g. confidence intervals)
☐	☒	For null hypothesis testing, the test statistic (e.g. *F*, *t*, *r*) with confidence intervals, effect sizes, degrees of freedom and *P* value noted<br>*Give P values as exact values whenever suitable.*
☐	☒	For Bayesian analysis, information on the choice of priors and Markov chain Monte Carlo settings
☒	☐	For hierarchical and complex designs, identification of the appropriate level for tests and full reporting of outcomes
☐	☒	Estimates of effect sizes (e.g. Cohen's *d*, Pearson's *r*), indicating how they were calculated

Our web collection on statistics for biologists contains articles on many of the points above.

Software and code

Policy information about availability of computer code

Data collection

For the collection of pupil data during Experiments 1, 2 and 3, we used MATLAB version R2020a (Exp1), or 2013a (Exp2 and 3) and the Tobii Pro Nano (Exp1) or Tobii_TX300 SDK (Exp 2 and 3); for response recording of behavioral data, we used the MATLAB-based presentation software Psychtoolbox 3.0.17 (Exp 3). For the collection of the motor evoked potentials in Exp1, we used a surface electromyography (EMG) electrode (Bagnoli™ DE-2.1 EMG Sensors, Delsys, Inc.); Raw signals were amplified (sampling rate, 5 kHz), digitized with a CED micro 1401 AD converter and Signal software V2.13 (both Cambridge Electronic Design, Cambridge, UK), and stored on a personal computer for off-line analysis. The timing of the transcranial magnetic stimulation pulses and EMG data recording was controlled by MATLAB R2020a (MathWorks, Inc., Natick, MA, USA) connected to the CED via a custom microcontroller. For the recording of cardiovascular data in Exp1 we used a wireless, Bluetooth-based, wearable Shimmer3 system (© Shimmer 2017TM, Realtime Technologies Ltd) and the accompanying Shimmer MATLABTM Instrument Driver 2.8a; for the recording of cardiovascular and respiratory data in Experiment 2 and 3, we used the Biopac MP 160 system with the accompanying AcqKnowledge software version 5.0. For the recording of EEG data in Exp 2 and 3 we used64 gel-based Ag/AgCl active surface electrodes (Brain Products, Munich, Germany) placed in the actiCAP SNAP holders (Brain Products) according to the international 10-20 system. Raw signals (sampling rate, 1 kHz) were amplified with the actiCHamp Plus amplifier and the BrainVision Recorder software (Brain Products, Munich, Germany); Impedances were kept below 20 kOhm.

Data analysis

For (pre-)processing of pupil data, open-source software from Kret & Sjak-Shie (2018) and Matlab code (2018a or R2020a) (e.g., for baseline correction) was used. For (pre-) processing of cardiovascular data, we used the Matlab-based toolbox physiozoo (Matlab version 2021a) and Matlab code (Matlab version 2018a and R2020a). For (pre-) processing of electromyography data we used Matlab code (Matlab version R2020a). EEG data were preprocessed in MATLAB (version R2022a) with the EEGLAB Toolbox (version 2020.0), the Automagic preprocessing pipeline, and the spectral parameterization toolbox FOOOF (version 1.0.0; for Python 3.8) in combination with custom codes written in MATLAB. For all statistical analyses we used IBM SPSS version 28; R version 4.1.2/4.2.2 including the packages WRS2 (version 1.1-3), cocor

(version 1.1-4), and rmcorr (version 0.4.5, 0.6.0), JASP version 0.16.2, the Matlab-based SPM-1D toolbox (M.0.4.8), and the Fieldtrip toolbox (version 2022.12.12) .

For manuscripts utilizing custom algorithms or software that are central to the research but not yet described in published literature, software must be made available to editors and reviewers. We strongly encourage code deposition in a community repository (e.g. GitHub). See the Nature Portfolio guidelines for submitting code & software for further information.

Data

Policy information about availability of data

All manuscripts must include a data availability statement. This statement should provide the following information, where applicable:

- Accession codes, unique identifiers, or web links for publicly available datasets
- A description of any restrictions on data availability
- For clinical datasets or third party data, please ensure that the statement adheres to our policy

Source data for the main figures in the manuscript are openly available in the ETH Zurich Research Collection under https://doi.org/10.3929/ethz-b-000732589. Raw data that support the findings of this study cannot be made publicly available to protect participants' rights according to Swiss human research law. Individual participant data can be accessed by investigators who provide proof of relevant ethical approval for the intended analysis and fulfill data protection measures according to Swiss legal requirements. Requests to gain access to the raw data can be sent to the corresponding author.

Research involving human participants, their data, or biological material

Policy information about studies with human participants or human data. See also policy information about sex, gender (identity/presentation), and sexual orientation and race, ethnicity and racism.

Reporting on sex and gender	The reported results apply to the female and male sex: our sample included male and female participants. This information was determined based on self-reports of the participants.<br>We did not conduct sex-/gender-based analyses in addition to our group level analyses since our study included both sexes and was not powered to conduct separate analyses
Reporting on race, ethnicity, or other socially relevant groupings	We did not conduct race/ethnicity-based analyses in addition to our group level analyses since our study was not powered to conduct separate analyses.
Population characteristics	See behavioral and social sciences study design information.
Recruitment	Healthy participants of the present study were recruited via online advertisement on University web pages. We are not aware of any self-selection bias, however, one bias that may impact the results is that the majority of the participants were young healthy university students in Switzerland. All participants gave written informed consent.
Ethics oversight	All experimental protocols were approved by the research ethics committee of the canton of Zurich (KEK-ZH 2018-01078)

Note that full information on the approval of the study protocol must also be provided in the manuscript.

Field-specific reporting

Please select the one below that is the best fit for your research. If you are not sure, read the appropriate sections before making your selection.

☐ Life sciences ☒ Behavioural & social sciences ☐ Ecological, evolutionary & environmental sciences

For a reference copy of the document with all sections, see nature.com/documents/nr-reporting-summary-flat.pdf

Behavioural & social sciences study design

All studies must disclose on these points even when the disclosure is negative.

Study description	The present study is a quantitative experimental study.
Research sample	Participants were mainly healthy university students (undergraduate, graduate students) from different Universities in Zürich/ Switzerland. Since it's mainly including young adults the sample is not representative considering the general population<br>In the Experiment 1, participants were 26.2 +/- 4.96 years old (mean +/- SD), 11 female and 4 male.<br>In the Experiment 2, participants were 26.7 +/- 5.00 years old (mean +/- SD), 12 female and 11 male.<br>In the Experiment 3, participants were 25.6 +/- 5.92 years old (mean +/- SD), 12 female and 7 male.
Sampling strategy	In the Experiment 1, the required sample size was estimated based on a pilot experiment (N=7; Cohen's dz=1.04), using a power analysis (G*Power version 3.1). It revealed that 15 participants should be included to detect an effect of pupil size self-regulation on MEP amplitude with a two-tailed t-test (difference between upregulation to downregulation condition, normalized to baseline), alpha = 0.05, and 95% power.<br>In the Experiment 2, the sample size was similar to or larger than in previous experiments investigating the spectral slope as an

electrophysiological marker of arousal levels in humans.
In the Experiment 3, the sample size was similar to or larger than previous investigations of evoked EEG and pupillary responses.

Data collection	We used eye trackers (Tobii Pro Nano in Exp. 1; Tobii TX300 in Exp. 2 and 3) for the collection of pupil data, a single-pulse monophasic TMS was delivered using a 70mm figure-of-eight coil connected to the Magstim 200 stimulator (Magstim, UK) and electromyography (Bagnoli™ DE-2.1 EMG Sensors, Delsys, Inc.) was used for muscle data collection in Exp. 1; Bluetooth-based, wearable Shimmer3 ECG system (© Shimmer 2017TM, Realtime Technologies Ltd) was used in Exp1 and ECG, a peripheral pulse sensor and a respiratory belt (Biopac MP 160 system for Exp. 2 and 3) to collect heart rate and respiratory data. For the recording of EEG data in Exp 2 and 3 we used64 gel-based Ag/AgCl active surface electrodes (Brain Products, Munich, Germany) placed in the actiCAP SNAP holders (Brain Products) according to the international 10-20 system. Raw signals (sampling rate, 1 kHz) were amplified with the actiCHamp Plus amplifier and the BrainVision Recorder software (Brain Products, Munich, Germany); Impedances were kept below 20 kOhm. Behavioral data was acquired and stored offline on a PC. Demographic data and questionnaire data (e.g., for exclusion criteria) were acquired using paper and pencil. During data acquisition, no-one except for researchers and participants were present. Furthermore, during Exp.1 the subject and the researcher were separated by a partition wall and in Exp. 2 and 3, participants were in a Faraday cage with the experimenter being in the control room, thus participants could focus on themselves without the feeling of someone watching/influencing them during the experiments. Participants were blind regarding the hypotheses of the study; In Exp. 1 the screen of the participant was covered with a customed blind, and the experimenter was blind to the experimental conditions while delivering TMS pulses; the researchers analyzing the data were blind to the experimental condition/groups during preprocessing of all data.
Timing	In the Experiment 1, the data collection started in November 2022 and finished in February 2023. Data for Experiment 2 was collected from July 2022 to October 2022. In the Experiment 3, data acquisition started in July 2020 and finished in October 2021. This data acquisition phase (Exp. 3) also entails the initial training of participants, already published in Meissner et al. (2023).
Data exclusions	In Exp. 1 four participants had to be excluded: one because of technical problems with data acquisition, and three because of our analysis exclusion criteria (i.e., data from participants who showed differences in root mean square bgEMG > 0.001mV between pupil size upregulation and downregulation conditions were removed to avoid the possibility that the differences in bgEMG could drive the effects on MEP amplitude; note, that this exclusion criterion is especially conservative, as bgEMG itself could be influenced by successfully modulated arousal level).<br>In Exp. 2 two participants had to be excluded: one for personal reasons, one for difficulties in adhering to the study procedures. For ECG analyses, one additional participant needed to be excluded due to technical problems during ECG data acquisition.<br>In Exp. 3 three participants had to be excluded: two because of technical problems with pupil data acquisition, and one because of a recording issue with the EEG data. A large number of trials had to be excluded for additional two participants for the analyses of the ERPs, we report these analyses in the supplementary material.
Non-participation	No participant has declined participation after recruitment.
Randomization	Participants were not allocated to different groups. All experiments followed a within-subjects design.

Reporting for specific materials, systems and methods

We require information from authors about some types of materials, experimental systems and methods used in many studies. Here, indicate whether each material, system or method listed is relevant to your study. If you are not sure if a list item applies to your research, read the appropriate section before selecting a response.

Materials & experimental systems

n/a	Involved in the study
☒	☐ Antibodies
☒	☐ Eukaryotic cell lines
☒	☐ Palaeontology and archaeology
☒	☐ Animals and other organisms
☒	☐ Clinical data
☒	☐ Dual use research of concern
☒	☐ Plants

Methods

n/a	Involved in the study
☒	☐ ChIP-seq
☒	☐ Flow cytometry
☒	☐ MRI-based neuroimaging

Plants

Seed stocks	n/a
Novel plant genotypes	n/a
Authentication	n/a
