# Supplementary material for: Modulating cortical excitability and cortical arousal by pupil self-regulation
Source: Nat Commun. 2025 May 16;16:4552. doi: 10.1038/s41467-025-59837-5 (PMC12084627; doi:10.1038/s41467-025-59837-5)
Supplement: Supplementary file 3 — Transparent Peer Review file [file 41467_2025_59837_MOESM3_ESM.pdf]

nature portfolio

Peer Review File

Modulating cortical excitability and cortical arousal by pupil self-regulation

Corresponding Author: Dr Marieke Weijs

This file contains all reviewer reports in order by version, followed by all author rebuttals in order by version.

Version 0:

Reviewer comments:

Reviewer #1

(Remarks to the Author)
In three different studies, Weijs et al. probe different conceptions of “cortical excitability” and their relationship with physiological arousal. During EEG measurements, trained participants self-regulate arousal through pupil-based neurofeedback. Whereas one study focuses on spectral EEG measures during self-regulation, another one additionally presents TMS pulses to measure changes in motor-evoked potentials (MEP), and the third uses an auditory oddball task to compare event-related responses to standards and deviants during self up- vs downregulated arousal. They report that all three measures are sensitive to the different self-regulation states and propose that this could be an interesting avenue for neurological interventions.

Overall, this is an excellent collection of studies that makes clever use of the pupil-based neurofeedback approach that the lab has introduced to shine lights on arousal-excitability links from different angles, and that should be of great interest - and bridges - several research communities. The evidence presented is satisfying and provides a unified picture. Applied methodology seems state of the art and fit for purpose. I still have a few comments after reading that the authors may want to consider:

Conceptually, I was hoping to read more about the mechanisms behind the pupil-based neurofeedback. Why/how do the authors think this approach works to influence excitability? It’s briefly alluded to that participants use different strategies to achieve up/down-regulated states but I’d encourage the authors to speculate a bit more.

Structurally, heart rate and heart rate variability are part of the analysis but this appears rather surprisingly, is not motivated in the introduction in detail, and no hypotheses have been formulated around that. Are these simply used as control measures due to their known links with arousal?

One methodological point: When splitting participants into groups based on their strategies for the MEP analysis, the most relevant test to see whether the group matters would be an ANOVA to check for a group x regulation state interaction. However, given the very small group sizes here (N=8 vs N=7) maybe a different approach that tests the influence of the strategy on a more continuous scale would be even better (- would it be possible to regress MEP difference, up vs down, from strategy, from more emotional to more physical?)

A final methods point: The relatively narrow and specific frequency range for the slope analysis (30-43 Hz) is not justified or motivated in the results, and using the full range (2-40Hz) only produces a “trend”. Can a better, more in detail justification be provided based on the existing literature? Otherwise this choice seems arbitrary.

Minor points:
Consider just using “pupil-based biofeedback” instead of the non-standard acronym “pupil-BF”. I read the “BF” as “basal forebrain” for a while.
Transition into the paragraph starting L85 can be improved for the reader
Line 204, briefly mention/introduce how slope was estimated
Line 218, correlation coefficient missing in brackets
Line 239, has the correct panel been referenced here?
Line 254, “which has been previously published” - repetition from a few lines up

Line 258, “spectral slope was significantly steeper” - for the same or a different frequency range as in Exp 2?
Line 279, statistical report unclear. “T” indicates t-test but looks more like the result of a non-parametric test?
Line 334, add reference to FOOOF paper (88)

Reviewer #2

(Remarks to the Author)
This study is valuable for two reasons:
- The authors recently published a paper in Nature Human Behaviour (https://pubmed.ncbi.nlm.nih.gov/37904022/) in which they describe a novel pupil-biofeedback method that allows individuals to voluntarily up- or downregulate their arousal. The results of the current paper provide further evidence for the power of this method.
- The authors use this method to show how pupil-linked baseline arousal is related to electrophysiological correlates of cortical excitability, cortical arousal and phasic LC activity. Some pharmacological and pupillometry evidence for these relationships already existed, but the current results add new ‘causal evidence’ for these relationships.
The results will be of interest to the large community of researchers who are interested in arousal and neuromodulation.

I have a number of concerns, mainly about how the results are presented, but I suggest possible solutions.

** Major comments **

1) To enable other researchers to use this novel method, it is very important that the authors share the exact protocol and instructions for participants of their pupil-based biofeedback method. I could not find this protocol in their previous publication, and I am a bit worried that they might not be willing/able to share this protocol, given the patent mentioned under 'Competing Interests'.

2. In my view, one of the findings is consistently overinterpreted in the manuscript: the linear relationship between pupil self-regulation strength and the modulation of the spectral slope. This correlation is based on a small number of participants (n=23), looks far from convincing (Fig. 5D), reaches a p-value that is only just significant (p = 0.048), and only *after* one outlier is removed from the scatterplot. I believe that this finding should be removed from the abstract and the final summary paragraph. The authors should also discuss this finding more cautiously on page 15. For a similar reason, I think that the paragraph about the other correlations (line 353 and further) should be toned down, and perhaps shortened. The sample sizes used in the three experiments are simply too small to allow firm conclusions concerning linear relationships between variables.

3) The frequency range used to determine the spectral slope, as discussed in the main text (30 to 43 Hz), seems rather arbitrary, especially the 43-Hz upper value. The authors state that this range was based on previous research, but the papers they cite used 30-45 or 30-50 Hz. How dependent are the conclusions of Experiment 2 on the upper value of this range?

4) I believe that the three main findings listed in the first paragraph of the discussion section should mirror the three main findings mentioned in the abstract: (i) MEP; (ii) spectral slope; (iii) P300. I do not think that the flattened spectral slope in response to target tones is one of the key findings; the study was not designed to investigate this, this finding may reflect artifacts, and there have already been several, more thorough studies of the effect of attention on spectral slope. I would also advise the authors to stress in this paragraph that they found similar pupil-regulation effects on spectral slope during task and rest.

** Minor comments **

Figure 2 caption: “After this modulation phase, participants received color-coded post-performance feedback (color coding: green in successful and pink in unsuccessful trials) …”. Perhaps be good to add here that this is feedback on the pupil modulation, not on oddball task performance.

Line 143: “(MD=.36, i.e., 36%)”. Here and elsewhere: what does MD mean and why is it followed by the same number as a percentage?

Line 147: remove word “highly”.
Line 216: remove word “strong”.

Line 217-218. “Including all participants, pupil size and spectral slope changes were largely unrelated (Pearson’s correlation: p=.70; Supplementary Figure 4c).” Something is wrong here: Supplementary Figure 4 shows a different p value (r = 0.42, p = .05) and different observations.

Line 256. Please indicate the frequency band used for these analyses (30-43 Hz is only mentioned in the figure caption).

Lines 567-568. “Each condition was repeated twice (i.e., a total of 6 blocks)”. What was the order of the conditions?

Lines 597, 766, 851: typos.

Lines 782-783. Common Average Reference and Independent Component Analysis should not be capitalized, even though the acronyms are capitalized.

Lines 943-944. “For the assessment of the P300, we defined a time window from 252-600ms after tone onset.” This time window seems arbitrary. What was it based on?

Lines 317-319. “…, it further supports the interpretation that the P300 may serve as an electrophysiological readout of phasic LC activity (34,44,45).” This would be a valuable reference to add: https://pubmed.ncbi.nlm.nih.gov/30232259/

Lines 350-352. Please rephrase sentence (e.g., what is meant with ‘both’?)

Line 363. Change ‘as’ to ‘than those’?

Line 394-395. Something is wrong with the language here.

Line 408-410. A highly relevant reference: https://pubmed.ncbi.nlm.nih.gov/38277436/

Lines 443-444. Two other relevant references: https://pubmed.ncbi.nlm.nih.gov/33186549/ and https://pubmed.ncbi.nlm.nih.gov/37367220/

Reviewer #3

(Remarks to the Author)
This study uses a previously established biofeedback-based self regulation of pupil dynamics in healthy participants and tests the impact of this manipulation on several markers of variations of central arousal state operating at different timescales: cortical excitability as measured through TMS-evoked muscle potentials, the slope of the aperiodic component of the EEG power spectrum, and the amplitude of P300 ERP components evoked by auditor oddball stimuli. The authors report that self-controlled up-regulation of pupil responses increase cortical excitability, flattens the decay of the EEG power spectrum with frequency, and reduces phasic arousal responses to oddball stimuli as measured by P300 (and conversely for down-regulating arousal). Up-regulating pupil-linked arousal also increases heart rate and reduces heart rate variability, two markers of peripheral arousal (more precisely: balance between sympathetic and parasympathetic tone). Individual variations in these two latter effects are unrelated to the effect on EEG spectral slope, while the effect on pupil diameter does correlate with the effect on EEG spectral slope. The latter points to a potentially important difference between markers of peripheral and central arousal, with pupil more strongly related to the latter.

I enjoyed reading this paper and think it can make an important contribution to the basic neuroscience literature, with plausible translational implications. The work builds immediately on a previous study from this team, published in Nature Human Behavior (Meissner et al, 2024), in both the methodology and part of the data. The current study is, however, original in analyzing complementary readouts of central arousal that were not assessed in the previous study.

That said, I have some concerns that should be addressed prior to publication.

Major:

1) Tone down overly specific conclusions about LC-origin of reported effects.
At various places, the authors equate their measurements (specifically: pupil diameter or P300 component) with actual LC-activity. E.g., in Abstract (almost identical statement in Discussion): "Here, we test whether pupil-based biofeedback modulates electrophysiological markers of cortical excitability, cortical arousal, and phasic LC activity." This is not warranted: authors did not measure LC activity, but the P300 component. The link between phasic LC responses and P300 has not been conclusively demonstrated, so this remains speculative. Similar issues pertain to the source of non-luminance mediated changes in pupil size. It is well-established that LC has a direct impact on those; but other brainstem nuclei (e.g. superior colliculi), including other neuromodulatory nuclei, have as well.
These statements may be intended as shorthands, since the authors are aware of this complexity as is evident from some statements in Abstract and a dedicated paragraph at the end of the conclusions. But they are scientifically inaccurate, and so I encourage them to refrain from such specific statements about LC involvement throughout the paper. Apart from the fact that such statements are not supported by the evidence presented, they are also not needed to to stress the significance of f this paper. The reported effects are sufficiently interesting and important, even if they were partly mediated by brainstem systems other than LC-NA.

2) Estimation of spectral slope.

The authors use a very narrow (and apparently arbitrary) range to estimate the slope of the aperiodic component of the EEG power spectrum: 30-40 Hz; when using a wider range (2-40 Hz), the effects are only marginally significant (Supplemental Figure 4). They motivate the narrow range by focus on a similar range in previous studies. In fact, only some of those used such a narrow range (Lendner et al, 2020), while more recent ones did use a much broader range (Pfeffer et al, 2022). These more recent studies, but not the older ones, were done without decomposition of the power spectrum using the FOOF algorithm -- which is relatively new, and which the authors also use here. If that algorithm works properly, there is no need to restrict the fit to such a narrow range, because the peaks in the <30 Hz range are fitted, and, theoretically, the aperiodic slope should explain the complete spectrum, down to a potential knee at very low frequencies.
The authors do not show the FOOF fits, so it is hard to evaluate if something may have gone wrong here. I encourage them to inspect this issue more closely, potentially improve their fitting procedure, etc. It would strengthen their case substantially if they could show that the pupil-linked changes in the spectral slope are robust with respect to the range, in which the fits were performed.

3) Is there a difference in correlation between effects of pupil, heart rate, or HRV with effects on spectral slope (Figure 5d)? As indicated about, the pattern of correlations (or lack thereof) shown in this Figure is very interesting. Please test for a difference in correlation: is the correlation between effects on pupil and spectral slope significantly larger than the respective correlations for effects on heart rate / HRV? This is important, because it could establish a physiological difference between markers of peripheral and central arousal.

Minor:

* The FOOF fits do not only provide estimates of the spectral slope of the aperiodic component, but also amplitude estimates of spectral peaks, specifically in the alpha-band. Did those also differ between pupil conditions? Did they correlate with the corresponding changes in spectral slope? Whether or not this is addressed, should be the authors' choice. I can just state that it would likely boost the broad impact of the paper because the relationship between pupil-linked arousal and cortical oscillations has been studied in some detail before, in rodents and humans (e.g., Vinck et al, Neuron, 2015; Pfeffer et al, eLife, 2022).

* Important methodological information about eye tracking seems to be missing in Methods. Which eye tracker and sampling rates were used, how did authors deal with fixational eye movements, etc.? Apologies if I may have missed this -- in that case, consider making this information more salient and moving it to the description of the subsection "Pupil-based biofeedback".

* It seems that the authors relate heart rate variability specifically to parasympathetic tone (line 231). Please elaborate on the rationale / evidence supporting this link. Why is heart rate variability not related to sympathetic tone?

Version 1:

Reviewer comments:

Reviewer #1

(Remarks to the Author)
The authors have fully addressed the issues I have raised in my first review. I have no further comments.

Reviewer #2

(Remarks to the Author)
The authors have carefully addressed my comments.

Reviewer #3

(Remarks to the Author)
The authors have done a thorough job in revising this manuscript, and have addressed all my concerns to my full satisfaction.

As for their analyses of the spectral components, which they comment on in their rebuttal: I leave it to the authors to decide whether to include those in the current paper or report it in a follow-up paper. The main thing is that this being communicated to the community, I because do think these results will be very relevant to develop a full picture of arousal dynamics in the brain.

Congratulations for this very interesting paper!

Open Access This Peer Review File is licensed under a Creative Commons Attribution 4.0 International License, which permits use, sharing, adaptation, distribution and reproduction in any medium or format, as long as you give appropriate credit to the original author(s) and the source, provide a link to the Creative Commons license, and indicate if changes were made.
In cases where reviewers are anonymous, credit should be given to 'Anonymous Referee' and the source.
The images or other third party material in this Peer Review File are included in the article's Creative Commons license, unless indicated otherwise in a credit line to the material. If material is not included in the article's Creative Commons license and your intended use is not permitted by statutory regulation or exceeds the permitted use, you will need to obtain permission directly from the copyright holder.
To view a copy of this license, visit https://creativecommons.org/licenses/by/4.0/

Reviewer #1 (Remarks to the Author):

In three different studies, Weijs et al. probe different conceptions of "cortical excitability" and their relationship with physiological arousal. During EEG measurements, trained participants self-regulate arousal through pupil-based neurofeedback. Whereas one study focuses on spectral EEG measures during self-regulation, another one additionally presents TMS pulses to measure changes in motor-evoked potentials (MEP), and the third uses an auditory oddball task to compare event-related responses to standards and deviants during self up- vs downregulated arousal. They report that all three measures are sensitive to the different self-regulation states and propose that this could be an interesting avenue for neurological interventions.

Overall, this is an excellent collection of studies that makes clever use of the pupil-based neurofeedback approach that the lab has introduced to shine lights on arousal-excitability links from different angles, and that should be of great interest - and bridges - several research communities. The evidence presented is satisfying and provides a unified picture. Applied methodology seems state of the art and fit for purpose. I still have a few comments after reading that the authors may want to consider:

1. Conceptually, I was hoping to read more about the mechanisms behind the pupil-based neurofeedback. Why/how do the authors think this approach works to influence excitability? It's briefly alluded to that participants use different strategies to achieve up/down-regulated states but I'd encourage the authors to speculate a bit more.

   We thank the reviewer for this suggestion. We have previously shown that pupil-based self-regulation modulates activity of the LC and other arousal regulating areas in the brainstem[1]. Crucially, we assume that up- and downregulation of pupil size leads to up- and downregulation of the LC-NA system, which generates testable hypotheses of how pupil-self regulation not only affects electrophysiological markers of arousal and the P300 but also cortical excitability as measured with single-pulse TMS. Furthermore, numerous studies show effects of pharmacological noradrenergic application on cortical excitability. This led to testable hypotheses of how pupil-based self-regulation affects cortical excitability. In the introduction, we present evidence on how pharmacological noradrenaline agents modulate cortical excitability and postulate how pupil-based self-regulation, which has been shown to be linked to a systematic modulation of the LC-NA system, can affect cortical excitability measured with single-pulse TMS evoked MEPs:

   Lines 55-63: "*First, numerous studies have provided causal evidence of noradrenergic contributions to changes in cortical excitability, as measured by transcranial magnetic stimulation (TMS) induced motor evoked potentials (MEPs). Specifically, administrating pharmacological NA application with TMS revealed that pharmacological noradrenergic enhancement via NA agonists increases the size of MEPs, indicating that enhanced NA has a facilitatory effect on cortical excitability*[15-22]*. Noradrenergic suppression, on the other hand, has been shown to lead to a decrease in excitability*[23]*. Such acute modulatory effects were observed after both single-dose and chronic drug administration and thus do not seem to depend on mechanisms specifically linked to long-term drug application (reviewed in*[24]*). (...)*"

   We now extended the description of the link between the evidence coming from previous studies to our research question to improve clarity:

Lines 93-101: *"Here, we aimed to further spotlight the effects of the recently developed pupil-based biofeedback approach on different markers of cortical excitability and arousal in a series of experiments using a multimodal approach (Figure 1a-e). Combining pupil-based biofeedback with single-pulse TMS, we investigated whether pupil self-regulation through pupil-based biofeedback modulates cortical excitability. In line with previous NA drug administration studies[25], we investigated whether pupil-based biofeedback, which has been shown to be linked to systematic up- and downregulation of the LC-NA system, can effectively modulate cortical excitability, similar to NA pharmacological agents. Specifically, we hypothesized to find increased acute cortical excitability reflected in larger MEPs when participants upregulate pupil size as compared to downregulate pupil size (Experiment 1, Figure 1c)."*

2. Structurally, heart rate and heart rate variability are part of the analysis but this appears rather surprisingly, is not motivated in the introduction in detail, and no hypotheses have been formulated around that. Are these simply used as control measures due to their known links with arousal?

   We thank the reviewer for pointing this out. The inclusion of these measures was motivated by their known link to arousal and evidence on noradrenergic projections from the locus coeruleus to parasympathetic and sympathetic control centers in the brainstem and spinal cord known to control cardiovascular functioning[2]. Well in line with this mechanistic link, our previous findings show that measures such as heart rate and heart rate variability are modulated through self-regulation of pupil size. We are afraid that a discussion of these mechanisms would be outside the scope of this manuscript, but we have included this additional reference in Line 55. We have now also included explicit hypotheses on hear rate and heart rate variability in the introduction.

   Introduction, lines 106-108: *"In line with our previous findings that self-regulation of pupil size also modulates cardiovascular arousal markers[14], we expected that HR would be higher, and HRV lower during pupil-size upregulation than downregulation."*

3. One methodological point: When splitting participants into groups based on their strategies for the MEP analysis, the most relevant test to see whether the group matters would be an ANOVA to check for a group x regulation state interaction. However, given the very small group sizes here (N=8 vs N=7) maybe a different approach that tests the influence of the strategy on a more continuous scale would be even better (- would it be possible to regress MEP difference, up vs down, from strategy, from more emotional to more physical?)

   We thank the reviewer for the suggestions. We initially did not expect any differences between "strategy subgroups", as the primary reason for categorizing participants into those using 'physical activation' versus 'emotional' mental strategies was to exclude the possibility that changes in motor cortex excitability were driven solely by motor imagery, as specified in lines 156-158. However, we appreciate the reviewer's suggestion to further investigate potential differences between the two subgroups. Our categorization of (i) purely emotional strategies and (ii) a mix of physical activation and emotional strategies is based on the debriefing after the experimental session. Although participants provided additional details on the specific strategies they used (e.g., the type of physical activity or emotion they imagined), it is not feasible to reliably transform these self-reports into a continuous scale required for regression analysis. Instead, we calculated the difference in MEP amplitude between up- and downregulation (normalized to the control rest

condition) for each subgroup (physical activation-related vs. emotion-related strategies) and compared these differences using an independent samples Bayesian t-test. We did not find a significant difference between the two subgroups and found anecdotal evidence for the H0. We have now added this to the manuscript.

Results, lines 164-167: "*The MEP amplitude, defined as the difference between up- and downregulation, normalized to control, did not differ between the groups using activation-related vs. emotion-related mental strategies (independent samples Bayesian t-test:* BF_{01}*=1.44, i.e. anecdotal evidence for H0).*"

Methods, lines 693-696: "*Additionally, to assess potential differences in MEP amplitude between the two groups (emotion-related vs. emotion- and activation-related), we calculated the difference in MEP amplitude between the up- and downregulation conditions, normalized it to the control rest condition, and compared the results using a independent samples Bayesian t-test.*"

4. A final methods point: The relatively narrow and specific frequency range for the slope analysis (30-43 Hz) is not justified or motivated in the results, and using the full range (2-40Hz) only produces a "trend". Can a better, more in detail justification be provided based on the existing literature? Otherwise this choice seems arbitrary.

We thank the reviewer for this valuable input. Our choice of frequency range of the slope analysis was based on previous literature, showing that especially frequency ranges between 30 and 50 Hz[3] and 30 and 45 Hz[4,5], respectively, reliably track changes in arousal level and show correlations with task performance. Similar to Höhn et al.[5], and Lendner et al.[4], we decided to stay below 45 Hz to avoid European line noise and, in our first approach, therefore implemented a 45 Hz low-pass filter. Due to the transition bandwidth (i.e., not a direct cut-off at 45 Hz), we fitted the slope for 30-43 Hz.

However, we now re-analyzed the data (i.e., Experiment 2 and Experiment 3), changed the low-pass filter to 60 Hz and implemented an additional notch filter to filter out the 50 Hz line noise. Then, we estimated the slope for 30-45 Hz[4,5] and performed all described analyses with the estimates of this slightly wider frequency range as previously reported in literature. The result patterns remain similar to the ones before (i.e., for a frequency range of 30-43 Hz), with (i) a significantly steeper slope during pupil size downregulation as compared to pupil size upregulation, an effect that was specific to the modulation phase, (ii) a significant correlation between differences in pupil size (i.e., up-down) and spectral slope (up-down; after outlier removal as described before) in Experiment 2, and (iii) a significantly steeper slope during pupil size downregulation as compared to pupil size upregulation and a counting control condition when participants performed a simultaneous oddball task in Experiment 3. Furthermore, as before, we observed a significant interaction between the sound (i.e., standard vs. target) and the time bin (i.e., BA vs. time bins after sound onset), which was driven by a flattening of the slope following the target but not the standard sounds. Finally, the repeated measures correlation between the spectral slope *prior to* target onset during the oddball task and the amplitude of the P300 to target tones was significant as reported before.

Respective changes in the Methods section:
Lines 801-805: "*For ERP analysis and the estimation of the spectral slope for a broader frequency range of 2-40Hz (see below for details), high- and low-pass filters were applied (i.e., high pass filter = 0.1Hz; low pass filter = 45Hz) to minimize slow drifts and trends and*

to eliminate 50Hz line noise. For the estimation of the spectral slope for 30-45 Hz (see below for details), the data was low pass filtered below 60Hz. Additionally, a notch filter (48-52Hz) was implemented to eliminate 50Hz line noise."

Lines 830-832: "*Based on previous studies, the frequency range was set to 30 to 45Hz (to ensure that no frequencies affected by filtering out European line noise were included) which has been shown to reliably differentiate different arousal states in humans[27] and E/I changes in animal work[26,27,29]."*

The results for Experiment 2 are reported in lines 213-231 and in Figure 5: "*As hypothesized, the spectral slope during downregulation was significantly steeper than during upregulation (two-sided paired samples t-test: 2.60; p=.016; d=.54; 95%-CI_d=[0.10;0.98]; Figure 5b); for similar trend-level results of the spectral slope estimated for 2-40Hz, see Supplementary Figure 4), suggesting a lower state of cortical arousal during downregulation as compared to upregulation of pupil size. Importantly, this difference was only present during self-regulation, especially 3-6s following self-regulation onset but not during baseline (rmANOVA: time bin*condition interaction: $F_{(3.08,67.71)}$=3.00, p=.035; η_p^2=.12; 95%-$CI\eta_p^2$=[0.00;0.24]; post-hoc paired samples t-tests: Baseline: p=.90; 0-3s: $t_{(22)}$=2.73; p=.06; d=.57; 95%-CI_d=[0.12;1.01]; 3-6s: $t_{(22)}$=2.94; p=.048; d=.61; 95%-CI_d=[0.16;1.05]; 6-9s: $t_{(22)}$=2.04; p=.16; d=.43; 95%-CI_d=[-0.01;0.85]; 9-12s: $t_{(22)}$=1.77; p=.18; d=.37; 95%-CI_d=[-0.06;0.79]; 12-15s: $t_{(22)}$=2.65; p=.06; d=.55; 95%-CI_d =[0.11;0.99]; corrected for multiple comparisons using sequential Bonferroni correction; Figure 5c), indicating a modulatory effect of pupil self-regulation on global cortical arousal measured with EEG. Including all participants, pupil size and spectral slope changes were largely unrelated (Pearson's correlation: r=-0.002, p=.99; Supplementary Figure 5a). However, this was mainly driven by an outlier with a very low pupil modulation index. Removing this outlier, we found a significant positive relationship between the pupil modulation index (upregulation-downregulation) and spectral slope changes (upregulation-downregulation; r=0.48; p=.025; 95%-CI_r=[0.07;0.75]; Figure 5d), thus better pupil self-regulation was associated with larger differences in spectral slope."*

Lines 242-247: "*Finally, we investigated whether changes in cortical arousal (i.e., the spectral slope, upregulation-downregulation) are directly linked with changes in cardiovascular arousal (i.e., heart rate (upregulation-downregulation) and RMSSD (downregulation-upregulation)). However, neither heart rate (all p>.20; Figure 5e, left panel) nor RMSSD changes (all p>.66; Figure 5e, right panel) showed a significant (linear) relationship with spectral slope changes, independent of the removal of the outlier (see pupil self-regulation modulates the spectral slope; Supplementary Figure 5)."*

The results for Experiment 3 are reported in lines 269-281 and Figure 6a,b*: "First, we investigated whether pupil self-regulation modulates the spectral slope between 30-45Hz during the auditory oddball task. As expected, the spectral slope was significantly steeper during downregulation as compared to upregulation and control trials, independent of the sound category (rmANOVA; main effect of condition: $F_{(2,36)}$=13.38; p<.001; η_p^2=0.43; 95%-$CI\eta_p^2$=[0.16;0.58]; post-hoc paired samples t-tests, corrected for multiple comparisons using sequential Bonferroni correction: upregulation vs. downregulation: $t_{(18)}$=-6.25; p<.001; d=-1.43; 95%-CI_d=[-2.07;-0.78]); upregulation vs. control: $t_{(18)}$=-.58; p=.57; d= -0.13; 95%-CI_d=[-0.58;0.32], Figure 6a); downregulation vs. control: $t_{(18)}$=3.57; p=.004; d=0.82; 95%-CI_d=[0.29;1.33]). Furthermore, this analysis revealed a significant time bin*sound interaction ($F_{(4,72)}$=3.51; p=.01; η_p^2=0.16; 95%-$CI\eta_p^2$=[0.01;0.28]). This interaction was driven by descriptively flatter slopes following target (i.e., 500-1000ms; $t_{(18)}$=2.72; p=.07; d=0.62; 95%-CI_d=[0.12;1.11]; after correction*

for multiple comparisons; Figure 6ab) but not prior to target as compared to standard sounds (p=.53). This effect was, however, independent of the self-regulation condition (i.e., no significant interaction effect with the factor condition; all p>.73)."

The updated results on the correlation between the spectral slope prior to target onset and the P300 are now reported in lines 311-316 and Figure 6g: *"Finally, we assessed whether the spectral slope prior to target sound presentation, representing the global cortical arousal state relates to the target-evoked P300 amplitude. A repeated-measures correlation between z-scored spectral slope prior to tone onset (-500-0ms) and z-scored P300 amplitudes in the 252-600ms time window across most prominent channels (Figure 6d) showed a significant negative correlation (r_{rm}=-.40, p=.01, 95%-CIr_{rm}=[-0.64;-0.10]; Figure 6g). Thus, a flatter slope (i.e., higher cortical arousal) at baseline relates to smaller P300 responses to oddball targets."*

The respective changes in the Discussion in lines 398-404: "*Here, we indeed found a significant modulation of the spectral slope during the auditory oddball task reflected by a significant sound (i.e., target vs. standard) * time bin (i.e., prior to sound versus after sound time bins) interaction: The spectral slope was descriptively flatter following the presentation of targets (i.e., in a time bin of 500-1000ms after tone onset; p = 0.07 after correction for multiple comparisons) as compared to standard sounds, suggesting that this flattening may be associated with attentional processes (Figure 6ab)."*

Minor points:

5. Consider just using "pupil-based biofeedback" instead of the non-standard acronym "pupil-BF". I read the "BF" as "basal forebrain" for a while.

   To avoid confusion, we removed the acronym and consistently use "pupil-based biofeedback" throughout the manuscript.

6. Transition into the paragraph starting L85 can be improved for the reader

   We have improved the transition, by highlighting the link of arousal and performance. Lines 77-79 now reads: *"Fluctuations in the brain's arousal state are tightly linked to task performance in an inverted U-shaped manner, suggesting an interplay between tonic activation patterns, reflecting the brain's global arousal state, and phasic responses related to task engagement and performance[2,34]."*

7. Line 204, briefly mention/introduce how slope was estimated

   We have included the following sentence, now in lines 211-213: *"The power spectral density was calculated for 3-s epochs during the pupil baseline and modulation phase and the aperiodic component of the power spectrum (i.e. spectral slope) was estimated for 30-45 Hz using the FOOOF toolbox[44]."*

8. Line 218, correlation coefficient missing in brackets

   We have added the correlation coefficient in line 226: "*Pearson's correlation: r=-0.002, p=.99.*" Please note that the correlation coefficient changed slightly (previous r = -0.08; p = 0.70) due to the changes in frequency range used for the estimation of the slope of the power spectrum (i.e., now from 30-45 Hz).

9. Line 239, has the correct panel been referenced here?

   Yes, the correct panel has been referenced here.

10. Line 254, "which has been previously published" - repetition from a few lines up

    We have removed this statement in line 266 to avoid unnecessary repetition.

11. Line 258, "spectral slope was significantly steeper" - for the same or a different frequency range as in Exp 2?

    We used the same narrowband frequency range from 30 – 45 Hz to estimate the spectral slope in Experiment 2 and 3. We have included the range in lines 269-270: *"First, we investigated whether pupil self-regulation modulates the spectral slope between 30-45 Hz during the auditory oddball task."*

12. Line 279, statistical report unclear. "T" indicates t-test but looks more like the result of a non-parametric test?

    This is the result of a cluster-based permutation t-test on the event related potentials, we now include this information in line 290: *"The P300 between the downregulation and upregulation condition also differed significantly (cluster-based permutation t-test: t=825.7, p=.036)."*

13. Line 334, add reference to FOOOF paper (88)

    We have added this reference (44) now in line 347.

Reviewer #2 (Remarks to the Author):

This study is valuable for two reasons:

- The authors recently published a paper in Nature Human Behaviour (https://pubmed.ncbi.nlm.nih.gov/37904022/) in which they describe a novel pupil-biofeedback method that allows individuals to voluntarily up- or downregulate their arousal. The results of the current paper provide further evidence for the power of this method.

- The authors use this method to show how pupil-linked baseline arousal is related to electrophysiological correlates of cortical excitability, cortical arousal and phasic LC activity. Some pharmacological and pupillometry evidence for these relationships already existed, but the current results add new 'causal evidence' for these relationships.

The results will be of interest to the large community of researchers who are interested in arousal and neuromodulation.

I have a number of concerns, mainly about how the results are presented, but I suggest possible solutions.

**** Major comments ****

1. To enable other researchers to use this novel method, it is very important that the authors share the exact protocol and instructions for participants of their pupil-based biofeedback method. I could not find this protocol in their previous publication, and I am a bit worried that they might not be willing/able to share this protocol, given the patent mentioned under 'Competing Interests'.

   We thank the reviewer for raising this important concern. The technical details on how pupil size is recorded and feedback is calculated are included in the Methods, section *Pupil-based biofeedback,* lines 493-546. In our previous publication in Nature Human Behaviour[1], we describe every step of the pupil-based biofeedback protocol and algorithm. The important steps are reiterated here, especially how post-trial feedback is calculated, because that is what we used in the reported experiments. Unfortunately, the code of the pupil-based biofeedback algorithm cannot be made publicly available since it is proprietary software of ETH Zurich and cannot be shared beyond the detailed description of the algorithm given in the methods section. However, researchers interested in verifying and reproducing our results can do so on location in a secured environment at the Neural Control of Movement Laboratory, ETH Zurich, upon signing a confidentiality agreement.

   Regarding exact instructions that participants receive, we explained to participants that they can use various strategies to increase and decrease the size of the signal they see on the screen as much as possible and provided some examples of such strategies. We now included this information in the Supplementary Material (Supplementary Table 1).

2. In my view, one of the findings is consistently overinterpreted in the manuscript: the linear relationship between pupil self-regulation strength and the modulation of the spectral slope. This correlation is based on a small number of participants (n=23), looks far from convincing (Fig. 5D), reaches a p-value that is only just significant (p = 0.048), and only *after* one outlier is removed from the scatterplot. I believe that this finding should be removed from the abstract and the final summary paragraph. The authors should also discuss this finding more cautiously on page 15. For a similar reason, I think that the paragraph about the other correlations (line 353 and further) should be toned down, and perhaps shortened. The sample sizes used in the three experiments are simply too small to allow firm conclusions concerning linear relationships between variables.

   We agree with the reviewer that we only had limited power to detect linear relationships between variables. We have removed this statement from the abstract and toned down the discussion section in lines 354-366.

   "The pupil modulation index (i.e., the difference between pupil size upregulation and downregulation) reflecting pupil self-regulation ability was linearly linked to differences in the spectral slope between up- and downregulation trials (Figure 5d). This observation is consistent with previous results reporting a tight relationship between pupil-based arousal measures and the spectral slope in humans, both during quiet wake[29] and non-rapid eye movement sleep stages[51]. Even though the interpretation of this correlation warrants caution due to a lack of statistical power to reliably detect linear relationships in the current study, it suggests that increases and decreases in pupil-linked arousal may parallel increases and decreases in E/I ratios in cortical neural circuits, respectively. This interpretation aligns with previous evidence that systematically activating the LC-NA system not only causes rapid pupil dilation in rodent models[12,13] but also that NA-enhancing drugs increase the E/I ratios in the cortex, as demonstrated by computational

modeling in humans[52]. Here we show that both pupil size and spectral slope are modulated via pupil self-regulation previously related to activity changes in the LC-NA system[14].”

3. The frequency range used to determine the spectral slope, as discussed in the main text (30 to 43 Hz), seems rather arbitrary, especially the 43-Hz upper value. The authors state that this range was based on previous research, but the papers they cite used 30-45 or 30-50 Hz. How dependent are the conclusions of Experiment 2 on the upper value of this range?

   We thank the reviewer for this valuable input. Similar to previous publications[4,5], we decided to stay below 45 Hz to avoid European line noise and, in our first approach, therefore implemented a 45 Hz low-pass filter. Due to the transition bandwidth (i.e., not a direct cut-off at 45 Hz), we fitted the slope for 30-43 Hz. However, we now re-analyzed the EEG data, changed the low-pass filter to 60 Hz and implemented an additional notch filter to filter out the 50 Hz line noise. Then, we estimated the slope for 30-45 Hz[4,5] and performed all described analyses with the estimates of this larger frequency range. This re-analysis showed that the results of Experiment 2 are not dependent on the upper value of the previous frequency range since the result pattern stays the same: Similar to the previous results on 30-43 Hz, we found a significantly steeper slope during pupil size downregulation as compared to pupil size upregulation, an effect that was specifically observed during the modulation but not the baseline phase. Furthermore, there was a significant correlation between differences in pupil size (i.e., up-down) and spectral slope (up-down; after outlier removal as described before).

   Respective changes in the Methods section of the manuscript:
   Lines 801-805*: “For ERP analysis and the estimation of the spectral slope for a broader frequency range of 2-40Hz (see below for details), high- and low-pass filters were applied (i.e., high pass filter = 0.1Hz; low pass filter = 45Hz) to minimize slow drifts and trends and to eliminate 50Hz line noise. For the estimation of the spectral slope for 30-45 Hz (see below for details), the data was low pass filtered below 60Hz. Additionally, a notch filter (48-52Hz) was implemented to eliminate 50Hz line noise.”*

   The results are now reported in lines 213-231 and in Figure 5: “*As hypothesized, the spectral slope during downregulation was significantly steeper than during upregulation (two-sided paired samples t-test: 2.60; p=.016; d=.54; 95%-CI$_d$=[0.10;0.98]; Figure 5b); for similar trend-level results of the spectral slope estimated for 2-40Hz, see Supplementary Figure 4ab), suggesting a lower state of cortical arousal during downregulation as compared to upregulation of pupil size. Importantly, this difference was only present during self-regulation, especially 3-6s following self-regulation onset but not during baseline (rmANOVA: time bin*condition interaction: $F_{(3.08,67.71)}$=3.00, p=.035; η_p^2=.12; 95%-CIη_p^2=[0.00;0.24]; post-hoc paired samples t-tests: Baseline: p=.90; 0-3s: $t_{(22)}$=2.73; p=.06; d=.57; 95%-CI$_d$=[0.12;1.01]; 3-6s: $t_{(22)}$=2.94; p=.048; d=.61; 95%-CI$_d$ =[0.16;1.05]; 6-9s: $t_{(22)}$=2.04; p=.16; d=.43; 95%-CI$_d$ =[-0.01;0.85]; 9-12s: $t_{(22)}$=1.77; p=.18; d=.37; 95%-CI$_d$=[-0.06;0.79]; 12-15s: $t_{(22)}$=2.65; p=.06; d=.55; 95%-CI$_d$ =[0.11;0.99]; corrected for multiple comparisons using sequential Bonferroni correction; Figure 5c), indicating a modulatory effect of pupil self-regulation on global cortical arousal measured with EEG. Including all participants, pupil size and spectral slope changes were largely unrelated (Pearson’s correlation: r=-0.002, p=.99; Supplementary Figure 5a). However, this was mainly driven by an outlier with a very low pupil modulation index. Removing this outlier, we found a significant positive relationship between the pupil modulation index*

(upregulation-downregulation) and spectral slope changes (upregulation-downregulation; r=0.48; p=.025; 95%-CI$_r$=[0.07;0.75]; Figure 5d), thus better pupil self-regulation was associated with larger differences in spectral slope."

Lines 242-247: "*Finally, we investigated whether changes in cortical arousal (i.e., the spectral slope, upregulation-downregulation) are directly linked with changes in cardiovascular arousal (i.e., heart rate (upregulation-downregulation) and RMSSD (downregulation-upregulation)). However, neither heart rate (all p>.20; Figure 5e, left panel) nor RMSSD changes (all p>.66; Figure 5e, right panel) showed a significant linear relationship with spectral slope changes, independent of the removal of the outlier (see pupil self-regulation modulates the spectral slope; Supplementary Figure 5)."*

4. I believe that the three main findings listed in the first paragraph of the discussion section should mirror the three main findings mentioned in the abstract: (i) MEP; (ii) spectral slope; (iii) P300. I do not think that the flattened spectral slope in response to target tones is one of the key findings; the study was not designed to investigate this, this finding may reflect artifacts, and there have already been several, more thorough studies of the effect of attention on spectral slope. I would also advise the authors to stress in this paragraph that they found similar pupil-regulation effects on spectral slope during task and rest.

   We thank the reviewer for this suggestion and have restructured the first paragraph of the discussion (lines 318-332) to highlight the three main findings and emphasize that we found a modulation in the spectral slope both during a pure self-regulation and in a dual task condition. We have copied the paragraph here:

   "In a series of experiments, we investigated whether pupil self-regulation, previously shown to tap into arousal-regulating centers in the brainstem including the LC-NA system[14], modulates previously suggested electrophysiological markers of cortical excitability, cortical arousal and the P300 response. Combining pupil-based biofeedback with (i) single-pulse TMS, (ii) EEG recordings, and (iii) a simultaneous auditory oddball task revealed three main findings: First, pupil self-regulation significantly modulated (i) cortical excitability measured with TMS (Figure 3). Second, pupil self-regulation significantly modulated the slope of the EEG power spectrum (i.e., spectral slope), an electrophysiological marker of cortical arousal in humans, proposed to reflect intrinsic excitability via capturing shifts in E/I ratios[26,27] both in a self-regulation only condition(Figure 5) and in a dual task setting where self-regulation was combined with an auditory oddball task (Figure 6a). Third, pupil self-regulation significantly modulated the P300 response elicited by target tones (Figure 6d-f). This corroborates our previous findings of larger pupil dilation responses during pupil size downregulation of the same dataset[14]. Based on our findings of the modulation of LC activity via pupil self-regulation, it further supports the interpretation that the P300 may serve as an electrophysiological readout of phasic LC activity[34,45–47]."

**** Minor comments ****

5. Figure 2 caption: "After this modulation phase, participants received color-coded post-performance feedback (color coding: green in successful and pink in unsuccessful trials) …". Perhaps be good to add here that this is feedback on the pupil modulation, not on oddball task performance.

We have included this information in the caption of Figure 2, the sentence now says: "*After this modulation phase, participants received color-coded feedback on their pupil regulation performance (color coding: …*".

6. Line 143: "(MD=.36, i.e., 36%)". Here and elsewhere: what does MD mean and why is it followed by the same number as a percentage?

   MD refers to 'mean difference'; we have now added the full term at its first occurrence in the text. Here, the mean difference is calculated between the two compared conditions (MEPs in up- vs. downregulation, normalized to the resting control condition). To improve clarity and avoid confusion, we have removed the percentage values.

7. Line 147: remove word "highly".

   We have removed "highly" in line 148.

8. Line 216: remove word "strong".

   We have removed "strong" in line 224.

9. Line 217-218. "Including all participants, pupil size and spectral slope changes were largely unrelated (Pearson's correlation: p=.70; Supplementary Figure 4c)." Something is wrong here: Supplementary Figure 4 shows a different p value (r = 0.42, p = .05) and different observations.

   We thank the reviewer for catching this wrong reference to Supplementary Figure 4c. Correlations between pupil size and spectral slope changes including all participants are shown in Supplementary Figure 5. We corrected this mistake in the manuscript in lines 225-227: "*Including all participants, pupil size and spectral slope changes were largely unrelated (Pearson's correlation: p=.99; Supplementary Figure 5a).*"

10. Line 256. Please indicate the frequency band used for these analyses (30-43 Hz is only mentioned in the figure caption).

    We have now included this information in lines 269-270: *"First, we investigated whether pupil self-regulation modulates the spectral slope between 30-45 Hz during the auditory oddball task."*

11. Lines 567-568. "Each condition was repeated twice (i.e., a total of 6 blocks)". What was the order of the conditions?

    The order of conditions was pseudorandomized and counterbalanced across participants. We now corrected the description in the manuscript, lines 588-589: "*Each condition was repeated twice (i.e., a total of 6 blocks), and the order of conditions was pseudorandomized and counterbalanced across participants.*"

12. Lines 597, 766, 851: typos.

    We thank the reviewer for catching the respective typos. We have corrected them now.

13. Lines 782-783. Common Average Reference and Independent Component Analysis should not be capitalized, even though the acronyms are capitalized.

    We have corrected this in lines 809-810.

14. Lines 943-944. “For the assessment of the P300, we defined a time window from 252-600ms after tone onset.” This time window seems arbitrary. What was it based on?

    Visual inspection of the ERP time course showed – both spatially and temporally – a spread out P300 response. Therefore, in line with Murphy et al.[6], we decided to use the 250-600ms time window for our analyses. The lower cutoff of 252 ms was determined by the sampling rate of 250 Hz. We now added the reference line 976 and included the following reasoning in the methods section:

    Lines 966-968: “*Visual observation of the scalp topographies suggested a widely distributed difference between conditions, both spatially and temporally, so we used cluster-based permutation t-tests to analyse the P300 across the full scalp.*”

15. Lines 317-319. “..., it further supports the interpretation that the P300 may serve as an electrophysiological readout of phasic LC activity (34,44,45).” This would be a valuable reference to add: https://pubmed.ncbi.nlm.nih.gov/30232259/

    We have added this reference (47) in line 332.

16. Lines 350-352. Please rephrase sentence (e.g., what is meant with ‘both’?)

    We have rephrased this sentence, now in lines 364-366 to the following: *“Here we show that both pupil size and spectral slope are modulated via pupil self-regulation previously related to activity changes in the LC-NA system.”*

17. Line 363. Change ‘as’ to ‘than those’?

    We have changed this in line 377.

18. Line 394-395. Something is wrong with the language here.

    We have rephrased this sentence (now lines 408-412), it now reads: *“It remains to be determined whether EEG spectra lack the sensitivity to capture changes in attention-related processes as a function of global arousal levels, or whether our study, originally designed to detect differences in global arousal levels and psychophysiological readouts such as pupil dilation and P300 responses, lacked sufficient power to detect such differences.”*

19. Line 408-410. A highly relevant reference: https://pubmed.ncbi.nlm.nih.gov/38277436/

    We have now included this reference (57) in line 427.

20. Lines 443-444. Two other relevant references: https://pubmed.ncbi.nlm.nih.gov/33186549/ and https://pubmed.ncbi.nlm.nih.gov/37367220/

    We have added these references (60, 10) to lines 458-463:

"Although the current literature predominantly supports non-luminance-related pupil size fluctuations to be closely linked to activity changes in the LC[10–13], other mechanisms may be modulating such fluctuations directly or indirectly[60]. Especially arousal-mediating cholinergic brain regions in the basal forebrain[10], but also dopaminergic nuclei such as the ventral tegmental area and substantia nigra[61], serotonergic regions such as the dorsal raphe nuclei[9,61], and hypothalamic orexin neurons[62] have been shown to be linked to pupil-linked fluctuations."

Reviewer #3 (Remarks to the Author):

This study uses a previously established biofeedback-based self regulation of pupil dynamics in healthy participants and tests the impact of this manipulation on several markers of variations of central arousal state operating at different timescales: cortical excitability as measured through TMS-evoked muscle potentials, the slope of the aperiodic component of the EEG power spectrum, and the amplitude of P300 ERP components evoked by auditor oddball stimuli. The authors report that self-controlled up-regulation of pupil responses increase cortical excitability, flattens the decay of the EEG power spectrum with frequency, and reduces phasic arousal responses to oddball stimuli as measured by P300 (and conversely for down-regulating arousal). Up-regulating pupil-linked arousal also increases heart rate and reduces heart rate variability, two markers of peripheral arousal (more precisely: balance between sympathetic and parasympathetic tone). Individual variations in these two latter effects are unrelated to the effect on EEG spectral slope, while the effect on pupil diameter does correlate with the effect on EEG spectral slope. The latter points to a potentially important difference between markers of peripheral and central arousal, with pupil more strongly related to the latter.

I enjoyed reading this paper and think it can make an important contribution to the basic neuroscience literature, with plausible translational implications. The work builds immediately on a previous study from this team, published in Nature Human Behavior (Meissner et al, 2024), in both the methodology and part of the data. The current study is, however, original in analyzing complementary readouts of central arousal that were not assessed in the previous study.

That said, I have some concerns that should be addressed prior to publication.

Major:

1. Tone down overly specific conclusions about LC-origin of reported effects. At various places, the authors equate their measurements (specifically: pupil diameter or P300 component) with actual LC-activity. E.g., in Abstract (almost identical statement in Discussion): "Here, we test whether pupil-based biofeedback modulates electrophysiological markers of cortical excitability, cortical arousal, and phasic LC activity." This is not warranted: authors did not measure LC activity, but the P300 component. The link between phasic LC responses and P300 has not been conclusively demonstrated, so this remains speculative. Similar issues pertain to the source of non-luminance mediated changes in pupil size. It is well-established that LC has a direct impact on those; but other brainstem nuclei (e.g. superior colliculi), including other neuromodulatory nuclei, have as well.

   These statements may be intended as shorthands, since the authors are aware of this complexity as is evident from some statements in Abstract and a dedicated paragraph at the end of the conclusions. But they are scientifically inaccurate, and so I encourage them

to refrain from such specific statements about LC involvement throughout the paper. Apart from the fact that such statements are not supported by the evidence presented, they are also not needed to to stress the significance of f this paper. The reported effects are sufficiently interesting and important, even if they were partly mediated by brainstem systems other than LC-NA.

We thank the reviewer for pointing this out. Indeed, these statements were used as shorthands and we did not intend to equate our outcome measures with LC activity. Therefore, we have revised the text and now avoid overinterpreting our hypothesis and findings in relation to LC-mediated effects. Specifically, we removed the statement from the abstract, and have rephrased the following passages:

Lines 109-112: *"... we examined whether pupil self-regulation during a classical two-tone oddball task modulates (1) the spectral slope reflecting global arousal and (2) the P300 response, an electrophysiological marker that is modulated by arousal and may relate to phasic LC responses."*

Lines 318-320: "*In a series of experiments, we investigated whether pupil self-regulation, previously shown to tap into arousal-regulating centers in the brainstem including the LC-NA system*[14]*, modulates previously suggested electrophysiological markers of cortical excitability, cortical arousal and the P300 response."*

Lines 427-430: *"Consistently, we have previously shown that pupil size downregulation, associated with relatively lower arousal and activity in brainstem nuclei including the LC, led to larger pupil dilation responses and better task performance than a counting control task and pupil size upregulation*[14]*."*

2. Estimation of spectral slope.

   The authors use a very narrow (and apparently arbitrary) range to estimate the slope of the aperiodic component of the EEG power spectrum: 30-40 Hz; when using a wider range (2-40 Hz), the effects are only marginally significant (Supplemental Figure 4). They motivate the narrow range by focus on a similar range in previous studies. In fact, only some of those used such a narrow range (Lendner et al, 2020), while more recent ones did use a much broader range (Pfeffer et al, 2022). These more recent studies, but not the older ones, were done without decomposition of the power spectrum using the FOOF algorithm -- which is relatively new, and which the authors also use here. If that algorithm works properly, there is no need to restrict the fit to such a narrow range, because the peaks in the <30 Hz range are fitted, and, theoretically, the aperiodic slope should explain the complete spectrum, down to a potential knee at very low frequencies. The authors do not show the FOOF fits, so it is hard to evaluate if something may have gone wrong here. I encourage them to inspect this issue more closely, potentially improve their fitting procedure, etc. It would strengthen their case substantially if they could show that the pupil-linked changes in the spectral slope are robust with respect to the range, in which the fits were performed.

   We thank the reviewer for this valuable comment. Our choice of frequency range for the analysis of the spectral slope was based on previous literature showing that especially frequency ranges of 30-50 Hz[3] and 30-45 Hz[4,5], respectively, but not necessarily broader ranges reliably track changes in arousal level and show correlations with task

performance. Similar to Höhn et al.[5], and Lendner et al.[4], we decided to stay below 45 Hz to avoid European line noise and, in our first approach, therefore implemented a 45 Hz low-pass filter. Due to the transition bandwidth (i.e., not a direct cut-off at 45 Hz), we fitted the slope for 30-43 Hz. However, to be consistent with previous publications[4,5], we now re-analyzed the data (i.e., Experiment 2 and Experiment 3), changed the low-pass filter to 60 Hz and implemented an additional notch filter to filter out the 50 Hz line noise. Then, we estimated the slope for 30-45 Hz[4,5] and performed all described analyses with the estimates of this larger frequency range. The result patterns remain similar to the ones reported before (i.e., 30-43Hz) with (i) a significantly steeper slope during pupil size downregulation as compared to pupil size upregulation, an effect that was specific to the modulation phase, (ii) a significant correlation between differences in pupil size (i.e., up-down) and spectral slope (up-down; after outlier removal as described before) in Experiment 2, and (iii) a significantly steeper slope during pupil size downregulation as compared to pupil size upregulation and a counting control condition when participants perform a simultaneous oddball task in Experiment 3. Furthermore, as before, we observed a significant interaction between the sound (i.e., standard vs. target) and the time bin (i.e., BA vs. time bins after sound onset).

We are nevertheless aware that other publications, i.e., Pfeffer et al.[7], use a broader frequency range to link changes in the spectral slope to oscillatory activity. We therefore added this estimation and the respective results to the Supplementary Material revealing only trend-level effects here. However, we did not observe knees in the data and the fitting values for the 2-40 Hz frequency range were relatively high (mean R2 = 0.98; SD = 0.04). Therefore, we believe that less consistent results for the broader frequency range from 2-40 Hz do not necessarily relate to methodological issues but rather reflects the sensitivity of higher frequency ranges (i.e., between 30 and 45/50 Hz) to changes in arousal levels of the brain[3,4]. This is also illustrated in Figure 5a, where the steepness of the slope for upregulation versus downregulation deviates more strongly from 35 Hz onwards.

We now added the respective information on the R^2 to the methods section of the manuscript in lines 839-841 for Experiment 2: "*The mean R^2 for the 30-45 Hz estimation was 0.96 (SD 0.04), and for the 2-40 Hz estimation 0.98 (SD 0.04) indicating a good fit.*"

The respective information for Experiment 3 in lines 948-949: "*… mean R^2 30-45 Hz estimation: 0.96; SD 0.06.*"

3. Is there a difference in correlation between effects of pupil, heart rate, or HRV with effects on spectral slope (Figure 5d)?

   As indicated about, the pattern of correlations (or lack thereof) shown in this Figure is very interesting. Please test for a difference in correlation: is the correlation between effects on pupil and spectral slope significantly larger than the respective correlations for effects on heart rate / HRV? This is important, because it could establish a physiological difference between markers of peripheral and central arousal.

   We thank the reviewer for this interesting suggestion. We have now calculated the difference between the correlations and have included these analyses in the results and methods sections. We did not observe any significant differences in correlation strength between central and peripheral measures of arousal, however there was a trend for a difference between pupil size and heart rate variability (RMSSD). This is interesting and might point to different sympathetic and parasympathetic influences on pupil size and

heart rate variability, respectively. We refrain from discussing this in the main manuscript, because we only observed a trend. However, future studies specifically designed to disentangle the effects of self-regulation on central and peripheral arousal would indeed be very interesting to further understand potentially different mechanisms.

Lines 875-877: *"To test whether there was a difference in strength between correlations of the spectral slope with the pupil modulation index, heart rate, and heart rate variability we used Meng, Rosenthal, and Rubin's z test via cocor for r*[94,95]*."*

Lines 248-252: "*To assess whether correlations between the spectral slope and indices of cardiovascular (i.e. heart rate and RMSSD) and central arousal (i.e. pupil modulation index), we compared the strength of the correlations. The correlation between spectral slope change and the pupil modulation index did not differ significantly in strength when compared to its correlation with heart rate change (z=0.71, p=.48, 95%-CI_z=[-0.35;0.75]) or change in RMSSD (z=1.78, p=.075, 95%-CI_z=[-0.03;0.95]).*"

Minor:

4. The FOOF fits do not only provide estimates of the spectral slope of the aperiodic component, but also amplitude estimates of spectral peaks, specifically in the alpha-band. Did those also differ between pupil conditions? Did they correlate with the corresponding changes in spectral slope? Whether or not this is addressed, should be the authors' choice. I can just state that it would likely boost the broad impact of the paper because the relationship between pupil-linked arousal and cortical oscillations has been studied in some detail before, in rodents and humans (e.g., Vinck et al, Neuron, 2015; Pfeffer et al, eLife, 2022).

   We thank the reviewer for this thought-provoking suggestion. Our general aim in the recent manuscript was to provide evidence on whether pupil self-regulation is associated with changes in the spectral slope as a recently suggested marker of cortical arousal and excitability as well as with changes in the P300. However, we are also aware that there are very interesting contributions in literature linking the aperiodic component of the power spectrum to oscillatory activity. Even though this was not the main focus of the current manuscript, we extracted the estimation of the spectral peaks and their amplitude for the up- and downregulation conditions. Interestingly, there seem to be some modulations of the periodic components (e.g., a shift in the alpha peak frequency which is higher during upregulation as compared to downregulation) during the modulation but not the baseline phase.

   We would like to run more in-depth analysis of oscillatory activity in different frequency bands and their link to pupil-derived measures (e.g., as described in Pfeffer et al.[7]), but fear that this will go beyond the scope of the current manuscript, which is already quite long and «result-rich». Thus, rather than including these additional analyses here, we would like to report the observed oscillatory activity and their link to pupil self-regulation in a separate manuscript dedicated to this topic. However, we are open to suggestions from the editor and reviewers on whether you would like to see the additional analysis of spectral peaks in the present manuscript.

5. Important methodological information about eye tracking seems to be missing in Methods. Which eye tracker and sampling rates were used, how did authors deal with fixational eye movements, etc.? Apologies if I may have missed this -- in that case,

consider making this information more salient and moving it to the description of the subsection "Pupil-based biofeedback".

We have moved this information from the experiment-specific subsections to the general Pupil-based biofeedback section, to make the information more salient. Different eye trackers were used for Experiment 1, and Experiment 2 and 3, respectively, but the sampling rate for all experiments was constant at 60 Hz.

Lines 541-544: *"Pupil size and gaze were measured with an eye tracker at 60 Hz (595 Pro Nano, Tobii Technology AG, Stockholm, Sweden in Experiment 1 Tobii tx300, Tobii Technology AG, Stockholm, Sweden in Experiment 2 and 3). At the beginning of each experimental session, the eye tracker was calibrated using a 5-point calibration technique."*

6. It seems that the authors relate heart rate variability specifically to parasympathetic tone (line 231). Please elaborate on the rationale / evidence supporting this link. Why is heart rate variability not related to sympathetic tone?

While heart rate variability (HRV) in general is influenced by both sympathetic and parasympathetic tone, the high frequency component of HRV mostly correlates with parasympathetic tone and low frequency fluctuations are mediated through the slower, sympathetic influence on HRV. HRV as measured here, with the RMSSD, is most likely indicative of parasympathetic rather than sympathetic activity, which requires longer measurement windows. Our experimental design, with only short modulation periods of 15s, allowed us to only analyse faster fluctuating influences on HRV. RMSSD, the HRV parameter we extracted, has been proposed as a reliable measure for windows as short as 10s[8]. This rationale is included in the Methods section of the manuscript:

Lines 703-709: "*Heart rate, reflecting cardiovascular dynamics controlled by an interplay between the sympathetic and parasympathetic nervous system, was calculated by dividing 60 through the respective R-R intervals of the modulation or rest (control) phase. Furthermore, we computed the root mean square of successive differences (RMSSD) calculated based on R-R-intervals. We chose RMSSD because it is relatively free from breathing influences[87] and can be computed for intervals as short as 10s[88,89]. It represents a beat- to-beat measure of HRV in the time domain that is assumed to give meaningful insights about parasympathetic activity of the autonomic nervous system."*

References

1. Meissner, S. N. *et al.* Self-regulating arousal via pupil-based biofeedback. *Nat. Hum. Behav.* **8**, 43–62 (2024).
2. Samuels, E. & Szabadi, E. Functional neuroanatomy of the noradrenergic locus coeruleus: Its roles in the regulation of arousal and autonomic function part II: Physiological and pharmacological manipulations and pathological alterations of locus coeruleus activity in humans. *Curr. Neuropharmacol.* **6**, 254–285 (2008).
3. Gao, R., Peterson, E. J. & Voytek, B. Inferring synaptic excitation/inhibition balance from field potentials. *NeuroImage* **158**, 70–78 (2017).
4. Lendner, J. D. *et al.* An electrophysiological marker of arousal level in humans. *eLife* **9**, e55092 (2020).

5. Höhn, C., Hahn, M. A., Lendner, J. D. & Hoedlmoser, K. Spectral Slope and Lempel–Ziv Complexity as Robust Markers of Brain States during Sleep and Wakefulness. *eneuro* **11**, (2024).
6. Murphy, P. R., Robertson, I. H., Balsters, J. H. & O'Connell, R. G. Pupillometry and P3 index the locus coeruleus-noradrenergic arousal function in humans: Indirect markers of locus coeruleus activity. *Psychophysiology* **48**, 1532–1543 (2011).
7. Pfeffer, T. *et al.* Coupling of pupil- and neuronal population dynamics reveals diverse influences of arousal on cortical processing. *eLife* **11**, e71890 (2022).
8. Munoz, M. L. *et al.* Validity of (ultra-)short recordings for heart rate variability measurements. *PLOS ONE* **10**, e0138921 (2015).
